# An estimated 5% of new protein structures solved today represent a new Pfam family

**DOI:** 10.1107/S0907444913027157

**Published:** 2013-10-12

**Authors:** Jaina Mistry, Edda Kloppmann, Burkhard Rost, Marco Punta

**Affiliations:** a European Molecular Biology Laboratory, European Bioinformatics Institute (EMBL–EBI), Wellcome Trust Genome Campus, Hinxton, Cambridge CB10 1SA, England; b Department of Bioinformatics and Computational Biology, Fakultät für Informatik, Technical University Munich, Garching, Germany; c New York Consortium on Membrane Protein Structure, New York Structural Biology Center, 89 Convent Avenue, New York, NY 10027, USA; d Sanger Institute, Wellcome Trust Genome Campus, Hinxton, Cambridge CB10 1SA, England

**Keywords:** Pfam families, structural coverage, protein-sequence space

## Abstract

High-resolution structural knowledge is key to understanding how proteins function at the molecular level. The number of entries in the Protein Data Bank (PDB), the repository of all publicly available protein structures, continues to increase, with more than 8000 structures released in 2012 alone. The authors of this article have studied how structural coverage of the protein-sequence space has changed over time by monitoring the number of Pfam families that acquired their first representative structure each year from 1976 to 2012. Twenty years ago, for every 100 new PDB entries released, an estimated 20 Pfam families acquired their first structure. By 2012, this decreased to only about five families per 100 structures. The reasons behind the slower pace at which previously uncharacterized families are being structurally covered were investigated. It was found that although more than 50% of current Pfam families are still without a structural representative, this set is enriched in families that are small, functionally uncharacterized or rich in problem features such as intrinsically disordered and transmembrane regions. While these are important constraints, the reasons why it may not yet be time to give up the pursuit of a targeted but more comprehensive structural coverage of the protein-sequence space are discussed.

## Abstract 2

This study uses the Pfam database to show that the sequence redundancy of protein structures deposited in the PDB is increasing. The possible reasons behind this trend are discussed.

## 1. The quest for structural coverage of the protein-sequence space

Detailed knowledge of the three-dimensional structure of protein chains, of the way in which they assemble into multimeric protein complexes and of their interaction with other molecules (such as DNA, RNA and small ligands) provides us with invaluable information about their molecular function. This information can give important insights into, for example, the effect of mutations such as those caused by some non-synonymous single-nucleotide polymorphisms or can impact significantly on our ability to design new drugs (Jorgensen, 2009 ▶).

The Protein Data Bank (PDB; *i.e.* the repository of all publicly available protein structures; Rose *et al.*, 2013 ▶; Velankar *et al.*, 2012 ▶) contains the structures of about 241 000 individual protein chains (as of 17 September 2013). This number can be contrasted with almost 42 million sequences in the UniProt Knowledgebase (UniProtKB, Release 2013_08; The UniProt Consortium, 2013 ▶). The gap between the numbers of available structures and available sequences, however, can be considerably reduced by the use of homology modelling. This technique allows us to computationally predict the three-dimensional coordinates of a previously structurally uncharacterized protein using information from the experimental structure of one or more of its homologues (Kiefer *et al.*, 2009 ▶; Pieper *et al.*, 2004 ▶). While homology models have lower resolution compared with the experimentally determined structures that they are derived from, they are of sufficient quality to be of use during drug discovery (Patny *et al.*, 2006 ▶; Cavasotto & Phatak, 2009 ▶).

The work presented here belongs to and offers an update to a line of studies that have addressed the question of how close we are to the goal of structurally covering the protein-sequence space; that is, of having the structure of a homologue for each (or, more realistically, most) of the protein sequences available (Chothia, 1992 ▶; Yan & Moult, 2005 ▶; Chandonia & Brenner, 2006 ▶; Levitt, 2007 ▶; Nair *et al.*, 2009 ▶).

## 2. Methods

### 2.1. PDB data

We downloaded release dates for PDB structures from the PDB website (http://www.rcsb.org) using the customized table download option. The download was performed on 18 March 2013; however, we excluded any entries released in 2013. Among the resulting entries we considered only those PDB chains that had a ‘true’ label in the file provided to us by the CATH team (personal communication, 14 March 2013). Chains with a ‘true’ label are those that CATH considers suitable for reliable domain classification (although not all of them have been classified by CATH as yet), according to the following constraints: (i) the experimental method employed for solving the structure is either NMR or X-ray crystallo­graphy with resolution ≤4.0 Å or a different experimental method with resolution ≤4.0 Å; (ii) the fraction of non-Cα atoms is ≥0.7; and (iii) the protein-chain sequence length is ≥40 residues. Additionally, as mentioned above, we only considered structures that had been released by the end of December 2012. This resulted in a total of 196 469 distinct PDB protein chains (belonging to 81 395 structures; see Supplementary Material^1^). 91.1% of these structures were solved by X-ray crystallo­graphy, 8.7% by NMR and 0.2% using other techniques.

Note that over time a certain number of PDB entries are superseded by corrected/improved versions and the new structures are assigned the (release) date on which they replaced the old entries. Other structures are simply withdrawn. This means that the entity and number of released structures per year are likely to change to a certain extent. It also means that the numbers of released structures are to some extent inflated in more recent years, *i.e.* years for which there has been less time for entries to be replaced or withdrawn.

Information about X-ray structures solved by molecular replacement was downloaded from the PDB website using the customized table download option.

### 2.2. Calculation of yearly Pfam family structural coverage

We used *pfam_scan* (Finn *et al.*, 2010 ▶) with the -clan_overlap option to run all PDB chain sequences that we considered (see §[Sec sec2.1]2.1) against all 14 831 Pfam 27.0 profile hidden Markov models (HMMs). The -clan_overlap option was used to ensure that for each PDB chain we retrieved matches to all families, including matches to multiple families within the same Pfam clan, when they occurred. This meant that a single PDB chain could structurally cover more than one Pfam family. The *pfam_scan* default option for the choice of significance thresholds was used, which applied the Pfam-defined family-specific gathering thresholds (GA) for establishing alignment significance. We used PDB chain sequences as found in the pdb_seqres file (downloaded 18 March 2013). The *pfam_scan* results together with the release dates of the structures extracted from the PDB (see §[Sec sec2.1]2.1) were used to calculate the number of families structurally covered per year (for the period 1976–2012, where 1976 was the first year for which released structures were available in the PDB). For each year, a Pfam family was considered to be structurally covered if at least one protein chain released in that year matched the family profile HMM with a significant alignment score.

Additionally, we calculated the number of newly structurally covered families per year. We started by calculating structurally covered families for 1976 as above. All families assigned to 1976 were removed from the family list. We calculated structurally covered families for 1977 from the remaining families and we then removed them from the list. The same procedure was repeated for all following years until 2012 (inclusive).

As an alternative way to calculate new structural coverage of Pfam families, we used the PDB chain sequence-to-Pfam family mapping provided by the *PDBfam* website (Xu & Dunbrack, 2012 ▶; http://dunbrack2.fccc.edu/ProtCiD/PDBfam/default.aspx). *PDBfam* is primarily based on alignments between Pfam family profile HMMs and consensus sequences for the PDB chains that are derived from *PSI-BLAST* profiles (Altschul *et al.*, 1997 ▶). This protocol allows more PDB chains to be mapped to existing families (0.8% of PDB sequences remain unmapped compared with 2.5% when using *pfam_scan*). Note however that, contrary to what we have implemented with *pfam_scan* (see above), *PDBfam* does not allow the same region of a PDB protein sequence to be assigned to more than one family per clan. As a consequence, although more PDB structures are assigned to Pfam families by *PDBfam*, the number of families that we classify as structurally covered is essentially the same with the two methods (6499 with *pfam_scan* *versus* 6496 with *PDBfam*). We downloaded the PDBfam.txt.gz file with the *PDBfam* mapping (version from 31 July 2013) that uses the list of families from Pfam release 27.0. The calculation of newly covered Pfam families per year was performed as described above, this time using *PDBfam* to associate PDB chains with Pfam families rather than the *pfam_scan* output. Note that we discarded any mapping to Pfam-B families present in the downloaded PDBfam file (*i.e.* only mapping to Pfam-A families was considered).

### 2.3. Clustering of PDB chains that do not match any Pfam family

We clustered PDB chain sequences that had no match to any Pfam family according to the following protocol. (i) We used each of the *N* unmatched chain sequences as a query in an all-against-all *phmmer* search. *N* was equal to 4815 when using *pfam_scan* to map PDB chains to Pfam families and to 1488 when using the *PDBfam* mapping. (ii) We stored in an *N* × *N* matrix *E* values for all sequence pairs for which *phmmer* returned an alignment with *E* value ≤ 0.001. (iii) We used the matrix calculated in (ii) as input to the clustering program *MCL* (Enright *et al.*, 2002 ▶). Structural coverage of the resulting clusters over the years was calculated in the same way as for Pfam families (see §[Sec sec2.2]2.2). Note that a considerable number of PDB sequences carry histidine-rich expression/purification tags that can cause *phmmer* to return significant matches between unrelated sequences. This in turn may lead *MCL* to place these proteins in the same cluster(s). To mitigate this problem, we attempted to remove the most significant part of such tags by monitoring the number of histidines found at the N- and C-termini of the PDB sequences. If we found ≥5 histidines in the first 12 and/or last 12 positions, we removed the first 12 and/or last 12 amino acids from the sequence.

### 2.4. Running *MCL*

We ran *MCL* as follows: (i) mcxload -abc file.abc --stream-mirror --stream-neg-log10 -stream-tf ‘ceil(200)’ -o file.mci -write-tab file.tab, where file.abc was the pairwise *E*-value matrix calculated from the *phmmer* runs (see §2.3); and (ii) mcl file.mci -I 2.3 -use-tab file.tab. Note that the --stream-mirror option symmetrizes *phmmer* *E* values for (*a*, *b*) *versus* (*b*, *a*) chain sequence pairs (the lower *E* value is selected for both pairs). The choice of the inflation parameter *I* influences the granularity of the clustering (with higher values of *I* corresponding to a higher number of clusters). We ran a simple experiment to estimate a reasonable level of granularity. We considered all PDB protein chain sequences released in 2012 that had at least one significant match to a Pfam family according to the *pfam_scan* assignment (a total of 21 342 sequences). We applied the clustering procedure described above. The only difference was that in order to account for the increased number of *phmmer* searches being performed, we considered only *E* values of ≤10^−4^ (this gives a similar number of estimated false positives). We ran *MCL* using different inflation values and compared the number of clusters we obtained with the number of Pfam families that were matched by these sequences. We found that an inflation value of *I* = 2.3 generated a number of *MCL* clusters (2451) that was comparable to the number of Pfam families (2454 using *pfam_scan*). We hence chose *I* = 2.3 for clustering all chain sequences with no current match in Pfam, assuming that such a value would generate a reasonable estimate of the number of Pfam families needed to additionally cover them. We used *I* = 2.3 for clustering unmatched PDB chains both when the PDB-to-Pfam mapping was calculated *via* *pfam_scan* and when it was provided by *PDBfam*.

### 2.5. Analysis of Pfam families with or without a structural representative in the PDB

Transmembrane helices, coiled-coil regions and disordered regions were predicted in the seed alignments of all Pfam families in Pfam 27.0. Coiled-coil regions were predicted using default parameters with *ncoils* (http://www.russelllab.org/cgi-bin/coils/coils-svr.pl). Transmembrane regions were predicted using *Phobius* (Käll *et al.*, 2004 ▶) with the default options. Disordered regions were predicted using *IUPred* (Dosztányi *et al.*, 2005 ▶) with the long option.

Pfam families in Pfam 27.0 were divided into two categories depending on whether or not they contained a structural member. In particular, families with a structural member were those that had a match to a least one PDB sequence according to *pfam_scan* (as described in §2.2). For families in each of these two categories, we determined the mean size of the families, the proportion of domains of unknown function (DUFs) and the proportion of residues predicted to be coiled coil, transmembrane or disordered in the seed alignments.

### 2.6. Analysis of human Pfam families

We downloaded (19 October 2012) the UniProtKB/Swiss-Prot-reviewed protein sequences for *Homo sapiens* (taxonomic identifier 9606; 20 234 sequences) from the UniProtKB website (http://www.uniprot.org/). The human sequences were searched against the Pfam 27.0 profile HMMs using *pfam_scan* with default parameters. The resulting Pfam families that matched were taken as the set of human Pfam families. Within this set, we calculated the proportion of families that had a structural member, using the *pfam_scan* results (described in §2.2) to determine which families contained a structural member. Families were predicted to be multispan trans­membrane domains if ≥50% of the seed members contained ≥2 predicted transmembrane helices.

## 3. The number of structures released in the PDB each year continues to increase

**Figure 1:**
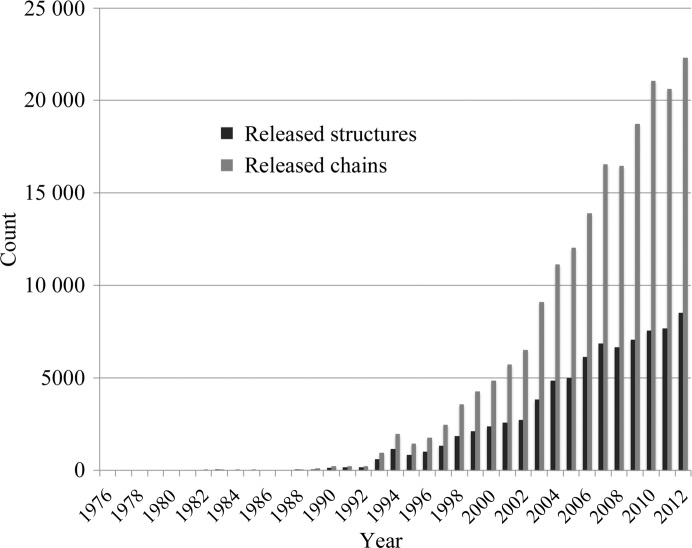
The number of structures (black) and the number of chains (grey) released each year in the PDB, from 1976 to 2012.

In Fig. 1 ▶, we show the numbers of structures and chains released in the PDB each year between 1976 and 2012 (for an account of recent PDB data-deposition trends, see Berman *et al.*, 2013 ▶). Note that we limited our analysis to a filtered list of PDB chains provided to us by the CATH database (Orengo *et al.*, 1998 ▶) team (§[Sec sec2]2). We found that the number of both yearly released structures and yearly released chains has increased over time. In 2012, 24% more structures and 35% more protein chains were released in the PDB than five years before in 2007; 211% and 242% more, respectively, were released than ten years before in 2002.

## 4. Analysis of PDB structures: from individual sequences to families

In order to better understand what the numbers reported in Fig. 1 ▶ mean in terms of progress towards more complete structural coverage of the protein sequence space, we considered PDB entries in the context of protein-sequence families (*i.e.* sets of homologous protein regions) and measured the increase in the number of families that are being structurally covered (*i.e.* that have at least one member with a known experimental structure). For this purpose we could use, in principle, the structure-based classification systems provided by SCOP (Andreeva *et al.*, 2008 ▶) or CATH (Orengo *et al.*, 1998 ▶). Using these resources, however, presents two problems. The first is that many of the structures released in recent years have not yet been included in the latest versions of SCOP and CATH (SCOP 1.75 and CATH v.3.5). The second is that by definition these databases only classify proteins for which structures have been solved. This means that they cannot provide us with any information on the number of protein families that are yet to be structurally characterized. To partially overcome these shortcomings, we decided to use the manually curated, mostly sequence-based Pfam database of protein families (Punta *et al.*, 2012 ▶). Pfam provides a higher coverage of PDB structures than either CATH or SCOP, and attempts to classify all protein regions, regardless of whether they fall into a family that contains a member whose structure has been characterized.

Each Pfam family has an associated seed alignment of representative protein regions and, built from this, a profile hidden Markov model (HMM) that can be used to search for further homologues in any collection of protein sequences. Profile HMMs are built using the *HMMER*3 suite of programs (http://hmmer.janelia.org/; Eddy, 2009 ▶). About 31% of Pfam families are grouped into clans (release 27.0); families in the same clan are believed to be evolutionarily related (Finn *et al.*, 2006 ▶). Homologous relationships between protein regions found in different families within a clan, however, are generally more remote than observed within a single family. For the purpose of this analysis, clan relationships were ignored.

It is important to note that Pfam families do not always correspond to structural domains. In particular, when sequence conservation extends seamlessly across more than one domain, such domains may end up as part of a single Pfam family. In other cases, the presence of weakly conserved regions within a domain may cause the domain to be split into two different Pfam families. Although structural information is used by Pfam to guide and improve family boundary definitions, this clearly does not apply to Pfam families for which no structural representative is available. Also, some Pfam families by definition do not represent structural domains. These include repeat and motif families (1.4% and 0.5% of the total families, respectively, in Pfam 27.0). The former are repeated structural elements that fold only when they occur in more than one copy along the protein sequence, *e.g.* the ankyrin (PF00023) and the WD40 (PF00400) repeats, while the latter are short conserved sequence motifs such as the AT-hook (PF02178) and the helix–hairpin–helix (PF00633) motifs. Finally, a few Pfam families cover intrinsically disordered protein regions (*e.g.* the human eukaryotic translation initiation factor 4E-binding protein 1; PF05456). Notwithstanding differences that may exist between Pfam families and structural domains, the Pfam classification provides a well founded means of studying sequence diversity in a protein data set. It was used, for example, as a source of novel domain targets by the Protein Structure Initiative (PSI-2; Chandonia & Brenner, 2005 ▶; Dessailly *et al.*, 2009 ▶).

Of all protein chains released in the PDB as of 2012, 97.5% can be mapped to at least one Pfam family (using *pfam_scan* and Pfam release 27.0; §[Sec sec2]2). This figure increases to 99.2% when the mapping is performed using profile–profile alignments as in *PDBfam* (Xu & Dunbrack, 2012 ▶; §[Sec sec2]2). The percentage of PDB residues that are found in a Pfam family is lower (80.6% when using *pfam_scan*). This is owing to the fact that chains with one or more Pfam matches may still have some regions/domains that are not yet classified in Pfam. Along the same lines, Pfam covers 80% of UniProtKB sequences (*i.e.* sequences with at least one Pfam match) and 58% of UniProtKB residues.

## 5. Latest trend: about five new protein families/clusters are structurally covered every 100 released structures

We ran *pfam_scan* for all 14 831 profile HMMs from Pfam release 27.0 against the collection of protein chain sequences available in the PDB at the end of 2012 (196 469 after filtering; see §[Sec sec2]2) and stored the significant Pfam matches for each sequence. We clustered the 2.5% of chains that returned no significant match to any profile HMM (4815 total) using the program *MCL* (Enright *et al.*, 2002 ▶; §[Sec sec2]2). Clusters obtained using *MCL* were used to provide a rough estimate of the actual number of families that will need to be built to include these chains into Pfam.

**Figure 2:**
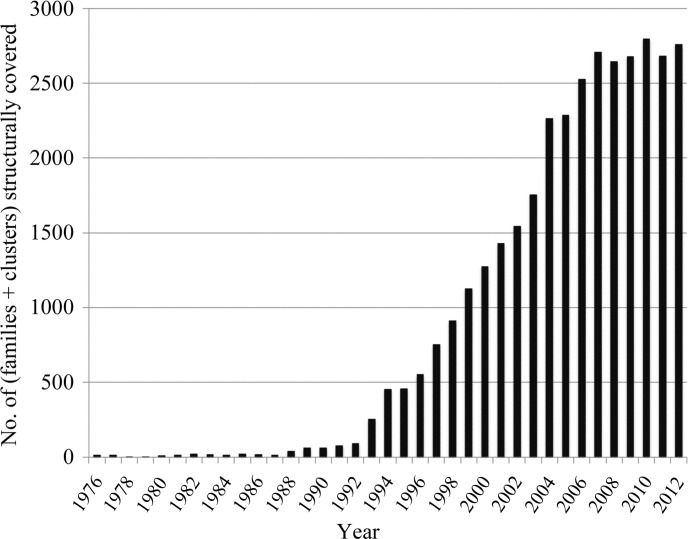
The number of structurally covered Pfam families and *MCL* clusters for each year between 1976 and 2012.

In the histogram in Fig. 2 ▶, the bars represent the number of Pfam families and *MCL* clusters that match structures released each year from 1976 to 2012 (Pfam families and *MCL* clusters are summed to give a single number for each year). We see that the number of families/clusters for which structures are solved each year seems to have reached a plateau at around 2600–2800. This is despite the growing number of released structures (Fig. 1 ▶). In 2007, 6858 structures were released which fell into 2705 Pfam families and *MCL* clusters; in 2012, 8507 structures (+24%) were released which fell into 2757 families and clusters (+2%). This means that according to this measure the sequence redundancy in the sets of structures that are released every year is increasing.

**Figure 3:**
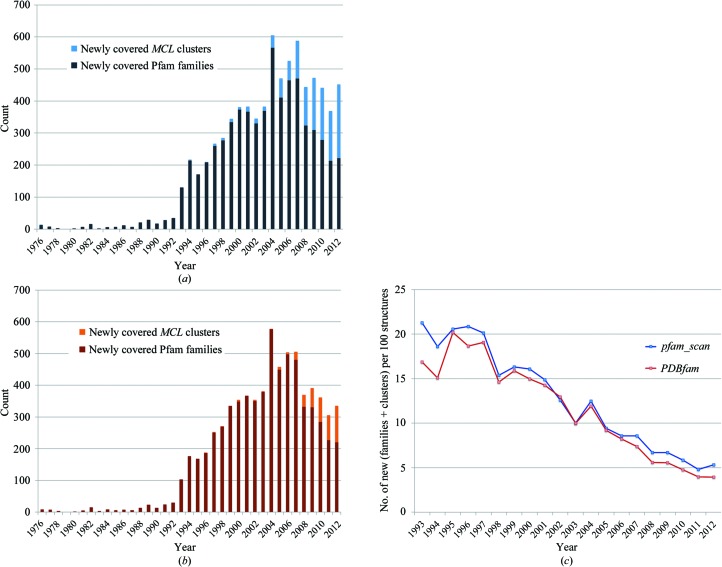
(*a*) The number of Pfam families (dark blue) and the number of *MCL* clusters (light blue) that gained their first structural representative each year from 1976 to 2012. *pfam_scan* was used to map Pfam families to PDB chains. (*b*) As in (*a*), but using *PDBfam* to map Pfam families to PDB chains. (*c*) The number of newly covered families/clusters (*i.e.* the sum of the two) per 100 structures released in the PDB each year from 1993 to 2012. Newly covered families/clusters were calculated using *pfam_scan* (blue) and *PDBfam* (red).

We now look at the figure that is of most interest to us, the number of families that acquired their first structural representative each year (newly structurally covered families). In Fig. 3 ▶(*a*), we show the number of newly structurally covered families for the period 1976–2012 (dark blue bars). Stacked on top of the families, we show the number of newly covered *MCL* clusters for the corresponding year (light blue bars). We see that the number of newly covered Pfam families/*MCL* clusters (*i.e.* the sum of the newly covered families and clusters) has remained relatively stable over the last five years at around 450. This is considerably fewer than achieved in 2004 and 2007 (605 and 588, respectively), and fewer, on average, than observed in the four years between 2004 and 2007 (an average of 436 *versus* an average of 547).

In Fig. 3 ▶(*b*), we show newly covered protein families and *MCL* clusters per year when using as an alternative method to assign PDB sequences to Pfam families the mapping provided by the PDBfam database. The trends appear similar to those observed when using the *pfam_scan* mapping.

Finally, we looked at the ratio between newly covered protein families/clusters and released PDB structures for each year from 1993 to 2012 , where 1993 was the first year in which more than 500 structures were released. In Fig. 3 ▶(*c*), we plot the number of newly covered families/clusters per 100 released structures. We see that over the last 20 years we have gone from about 20 to about five newly covered families/clusters per 100 released structures. Thus, at the current pace (Figs. 3 ▶ *a* and 3 ▶ *b*), achieving complete structural coverage for families that are currently in Pfam (release 27.0, 6499 families still to cover) would take an estimated 15–20 years.

## 6. Families that have no structural representatives include many families that are small, not functionally characterized or enriched in challenging protein regions

According to our analysis based on the Pfam classification, protein chains of newly solved structures ever more often fall into families that have a structural representative already (Fig. 3 ▶ *c*). In this section, we investigate the reasons behind this trend.

**Figure 4:**
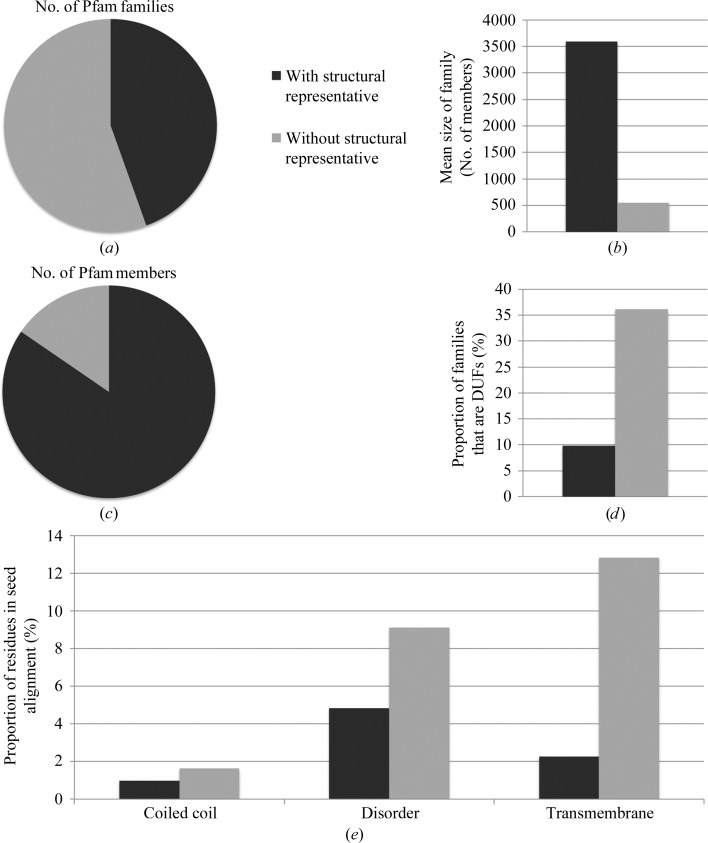
(*a*) Proportion of Pfam families that have a structural representative (using *pfam_scan*). (*b*) Mean size of Pfam families with and without a structural representative. (*c*) Proportion of Pfam family members that have a structural representative (*pfam_scan*). (*d*) Proportion (%) of Pfam families with and without a structural representative that are domains of unknown function (DUFs). (*e*) Proportion (%) of residues in the seed alignment of Pfam families, with and without a structural representative, that are predicted to be coiled-coil, disordered and transmembrane residues (see §[Sec sec2]2).

**Figure 5:**
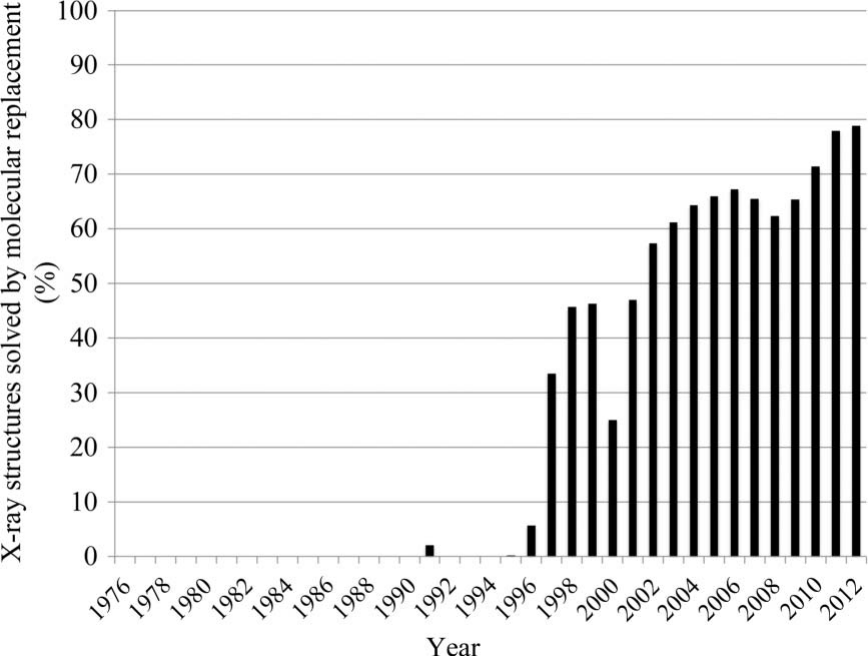
Proportion (%) of X-ray structures that have been solved using molecular replacement each year from 1976 to 2012.

A simple explanation would be that by now most of the sequence space could be structurally covered. A quick look at the list of Pfam families that still lack structural characterization would suggest that this is not the case. Of the 14 831 families that are part of Pfam release 27.0, just 44% have been structurally characterized as of 2012 (Fig. 4 ▶ *a*). Also, Pfam is growing. For example, the number of Pfam families increased from 13 672 in Pfam 26.0 (released in November 2011) to 14 831 in Pfam 27.0 (released in March 2013). A more detailed analysis of structurally uncharacterized Pfam families, however, shows that on average they are much smaller in size with respect to their structurally characterized counterparts (average number of 549 *versus* 3594 members; Fig. 4 ▶ *b*). Indeed, at the end of 2012, only 17% of all protein regions classified by Pfam fell into structurally uncharacterized families (Fig. 4 ▶ *c*). Thus, whenever targets are not specifically selected based on their sequence novelty, the fact that more and more newly solved structures fall into already characterized families is to be expected, as these families cover most of the sequence space currently classified by Pfam. This also has important consequences for the techniques used to solve new structures. Indeed, the percentage of X-ray structures that are solved each year by molecular replacement, a methodology that uses information derived from homologous proteins of known structure, is now approaching 80% (Fig. 5 ▶). It should be noted, however, that when applied to multi-domain proteins or to protein complexes, molecular replacement can also allow the structure of members of new Pfam families, *i.e.* members of families lacking a structural representative, to be determined.

On the other hand, family size is likely to not be the only reason behind the slow down in the structural characterization of novel Pfam families. We see that families that still lack structural representatives are enriched in domains of unknown function (DUFs; 36% compared with only 10% among families with structural representatives; Fig. 4 ▶ *d*). DUFs are families to which Pfam has not yet assigned any functional annotation. At the same time, these families are predicted (§[Sec sec2]2) to be enriched in coiled-coil, disordered and transmembrane residues (Fig. 4 ▶ *e*), which constitute regions that often make protein experimental structural characterization all the more challenging.

Finally, we should not forget that there are proteins that are relevant for increasing structural coverage of the protein-sequence space but that may not be very important/interesting in terms of their biology or their medical benefit. On the contrary, a number of already structurally characterized families and proteins are of special (*e.g.* biomedical) interest and are being studied in great detail by structural biologists. Some of these protein families, such as G-protein-coupled receptors (Venkatakrishnan *et al.*, 2013 ▶), are very functionally diverse and their members may interact with a number of different partners or adopt different conformational states. Structural data can go a long way in helping to characterize this variation if structures are determined for additional members of a family or for the same protein but under different experimental conditions. In Table 1 ▶, we report the ten most targeted Pfam families for 2012. We can see that during 2012 hundreds of structures were solved of protein kinases, immuno­globulins and trypsins.

## 7. Conclusions and (at least) one good reason not to give up on increasing the structural coverage of Pfam families

We have pointed to many valid reasons why structural biologists focus their efforts on trying to better characterize families and proteins for which at least some structural information is already available. We have also shown why a number of currently uncovered families appear to be ‘unpalatable’ for structure determination. While these considerations are important, we believe that there are still a considerable number of structurally uncharacterized families that are worth pursuing. Efforts have been made by some groups to identify such targets. One example is the JCSG structural genomics consortium, which is targeting structurally uncharacterized protein families that are overrepresented in metagenomic data (Ellrott *et al.*, 2010 ▶) and, more specifically, families in the human gut microbiome (some of these currently have very few members in UniProtKB). Also, a list of uncharacterized membrane proteins has recently been brought to the attention of researchers (Pieper *et al.*, 2013 ▶). Here, we add a brief perspective on structural coverage of the human proteome. For 90% of human sequences we have at least one match to a Pfam family (Pfam release 27.0). At the residue level, 45% of human residues fall into a Pfam region (Mistry *et al.*, 2013 ▶). Of all Pfam families with a match in the human proteome (5494 in total), 44% have no structural representative. If we remove families that are DUFs and families where the seed alignment contains ≥25% of residues that are predicted to be disordered, we are left with 1238 families that do not have a structural representative, of which 1003 are not predicted to be multispan transmembrane domains (§[Sec sec2]2). This is an example of a set of human families for which some functional information is in most cases already available (81% of them have at least one literature reference in Pfam) and for which solving the structure of a first member, if by no means easy (see also Bray, 2012 ▶), may be within reach of current structural determination techniques. A concerted effort to solve families such as these could yield a structural representative for most of them within the next few years.

## Supplementary Material

Excel spreadsheet with all PDB chains used in this study and their matches to Pfam families. Column 1 contains all PDB chains considered here (format is PDBidCHAINid); column 2 contains all pfam_scan matches for that PDB chain; column 3 contains all PDBfam matches for that PDB chain. The way matches are calculated is described in the Methods section of the paper.. DOI: 10.1107/S0907444913027157/ba5211sup1.xlsx

## Figures and Tables

**Table 1:** The ten families with the highest number of structures released in 2012 (families from Pfam release 27.0, matches according to *pfam_scan*; see §[Sec sec2]2) Note that if a structure had multiple chains that matched the same family then all of these matching chains counted as one structure in the last column of the table.

Pfam family accession No.	Pfam clan accession No.	Pfam family description	No. of structures released in 2012
PF00069	CL0016	Protein kinase domain	510
PF07714	CL0016	Protein tyrosine kinase	505
PF07654	CL0011	Immunoglobulin C1-set domain	227
PF07686	CL0011	Immunoglobulin V-set domain	196
PF13895	CL0011	Immunoglobulin domain	174
PF14531	CL0016	Kinase-like	145
PF08205	CL0011	CD80-like C2-set immunoglobulin domain	140
PF13927	CL0011	Immunoglobulin domain	137
PF00089	CL0124	Trypsin	128
PF00047	CL0011	Immunoglobulin domain	105

## Footnotes

1 Supplementary material has been deposited in the IUCr electronic archive (Reference: BA5211). Services for accessing this material are described at the back of the journal.
